# Acetic acid produced by *Staphylococcus epidermidis* remodels chromatin architecture and suppresses gene expression in *Malassezia restricta*

**DOI:** 10.1128/mbio.01592-25

**Published:** 2025-09-12

**Authors:** Jae Min Lee, Hyun Oh Yang, Hideki Tanizawa, Ken-ichi Noma, Tae Kwon Lee, Won Hee Jung, Yong-Joon Cho, Kyoung-Dong Kim

**Affiliations:** 1 Department of Systems Biotechnology, Chung-ang University, Anseong, Republic of Korea; 2 Institute for Genetic Medicine, Hokkaido University 89291, Sapporo, Japan; 3 Institute of Molecular Biology, University of Oregon, Oregon, USA; 4 Department of Environmental & Energy Engineering, Yonsei University 12810, Wonju, Republic of Korea; 5 Department of Molecular Bioscience, Kangwon National University 3265, Chuncheon, Republic of Korea; Dartmouth College Geisel School of Medicine, Hanover, New Hampshire, USA

**Keywords:** skin microbiome, acetic acid, chromatin architecture, *Malassezia*, *Staphylococcus*

## Abstract

The skin microbiome is composed of diverse microbial communities that engage in interkingdom interactions, influencing host physiology and microbial balance. Although *Malassezia restricta* and *Staphylococcus epidermidis* are codominant members of the human skin microbiome, the molecular mechanisms underlying their interactions remain poorly understood. We aimed to investigate the mechanism by which *S. epidermidis*-derived acetic acid affects chromatin organization and gene expression in *M. restricta. S. epidermidis* modulated chromatin structure and transcriptional activity in *M. restricta* by secreting acetic acid (AcOH), a common skin-associated organic acid. Using *in situ* Hi-C, we established the first three-dimensional genome architecture map of *M. restricta* and identified putative centromeric loci based on inter-chromosomal association scores. Co-culture with *S. epidermidis* or direct treatment with AcOH induced large-scale chromatin decompaction and enhanced centromeric clustering, indicating significant reorganization of the nuclear architecture. Through chromatin immunoprecipitation (ChIP)-seq analysis, we observed that AcOH exposure led to a redistribution of histone acetylation from promoter regions to gene bodies. This chromatin remodeling was further associated with extensive transcriptional repression, particularly of genes involved in translation, metabolism, and virulence, as revealed by RNA-seq analysis. Of note, these changes were specific to AcOH and were not replicated under inorganic acid stress (HCl), indicating a metabolite-specific epigenetic response. This study reveals a novel form of interkingdom communication in the skin microbiome, in which *S. epidermidis*-derived AcOH acts as an epigenetic modulator in *M. restricta*. Our findings provide key mechanistic insights into how bacterial metabolites influence fungal chromatin architecture and transcription, with implications for microbial community dynamics and skin health.

**IMPORTANCE:**

This study provides essential insights into interkingdom interactions within the human skin microbiome, highlighting how microbial metabolites influence fungal biology at the chromatin level. Specifically, we identify acetic acid (AcOH), secreted by *Staphylococcus epidermidis*, as a key regulator that induces significant chromatin remodeling and transcriptional changes in *Malassezia restricta*. By presenting the first three-dimensional genome architecture map of *M. restricta*, our findings uncover metabolite-specific chromatin dynamics that cannot be replicated by inorganic acid stress. Additionally, the conservation of this chromatin response in other *Malassezia* species suggests broader implications for understanding microbial adaptation mechanisms in the skin environment. This work underscores the critical role of bacterial metabolites as modulators of microbial interactions and provides new avenues for investigating microbial community balance and potential therapeutic strategies for skin health.

## INTRODUCTION

The human skin microbiome is a complex ecosystem comprising diverse bacterial and fungal species that coexist and interact to maintain skin homeostasis (1, 2). Among these microorganisms, *Malassezia* and *Staphylococcus* species constitute the dominant genera (3). *Malassezia* species are lipid-dependent yeast cells that inhabit sebaceous regions and have been implicated in various skin conditions, such as seborrheic dermatitis and atopic dermatitis (4, 5). *Staphylococcus epidermidis* is a commensal bacterium that contributes to skin barrier function, while *Staphylococcus aureus* is an opportunistic pathogen associated with inflammatory skin diseases (6, 7). Although the *Malassezia* and *Staphylococcus* genera have been extensively studied, the molecular mechanisms underlying their interkingdom interactions remain largely unexplored.

Recent studies have revealed intricate interkingdom interactions between *Malassezia* and *Staphylococcus* species that affect microbial growth, biofilm formation, and antifungal susceptibility (8–10). *Malassezia restricta* secretes aspartyl proteases that can influence biofilm formation in *S. aureus* (11), while *Staphylococcus* species produce metabolic by-products that alter *Malassezia* physiology (10). Among these microbial by-products, short-chain fatty acids produced by the skin microbiome, including acetic acid, butyric acid, and succinic acid, have been shown to influence microbial dynamics (12). Skin pH, which ranges from 4.5 to 5.5 and plays a key role in maintaining microbial balance, is a critical factor that influences microbial interactions (13, 14). *Staphylococcus* species contribute to skin acidification by secreting organic acids, primarily acetic acid (AcOH) and lactic acid. A previous study utilized high-performance liquid chromatography to quantify organic acids produced by *S. epidermidis* and *S. aureus* cultured under conditions identical to those used in the present study. Among the detected acids, AcOH was by far the most abundant, with concentrations nearly 10 times higher than those of lactic acid. Notably, *S. epidermidis* produced significantly greater amounts of AcOH compared to *S. aureus* (10). Although AcOH influences bacterial communities and fungal metabolism, its effects on fungal chromatin architecture and transcriptional regulation have not been explored.

Chromatin organization plays a crucial role in determining genome function as it influences transcriptional regulation, replication, and genome stability. In eukaryotic cells, chromatin is hierarchically structured, with higher-order organization defining transcriptional compartments and regulatory interactions. Nuclear architecture is associated with the formation of topologically associating domains (TADs), which segment the genome into distinct functional units that regulate gene expression (15, 16). Although chromatin architecture has been extensively characterized in yeast model systems such as *Saccharomyces cerevisiae* and *Schizosaccharomyces pombe* (17–21), the spatial organization of chromatin in *Malassezia* species is poorly understood. *M. restricta* is a dominant fungal species of the human skin and has been implicated in conditions ranging from dandruff to systemic infections (22, 23). Despite its clinical relevance, the mechanisms by which it organizes its genome or responds to environmental stress at the chromatin level remain unclear.

Histone acetylation is a key epigenetic modification that regulates chromatin accessibility and gene expression (24). H3K9 and H3K27 acetylation are associated with active transcription, whereas their deacetylation promotes chromatin compaction and gene repression (25, 26). Environmental factors, such as pH changes and microbial metabolite levels, have been shown to affect histone modifications, leading to chromatin reorganization and transcriptional shifts in fungi (27). Although acetate affects histone acetylation in mammalian and plant systems (28, 29), its role as an epigenetic regulator in skin-associated fungi remains unknown.

In this study, we aimed to investigate the mechanism by which *S. epidermidis*-derived AcOH affects chromatin organization and gene expression in *M. restricta*. Using an integrative multi-omics approach, including *in situ* Hi-C, chromatin immunoprecipitation (ChIP)-seq, and RNA-seq, we established the first three-dimensional genome architecture map of *Malassezia* and demonstrated that AcOH induces chromatin decompaction and histone acetylation redistribution and suppresses key transcriptional programs. Our study identified AcOH as an interkingdom epigenetic signaling molecule and reveals a novel mechanism by which bacterial metabolites influence fungal nuclear organization and function. Furthermore, this study expands our understanding of microbial crosstalk in the skin and highlights the consequences of interspecies metabolic interactions at the chromatin level.

## MATERIALS AND METHODS

### Strains and media

*M. restricta* KCTC 27527, *Malassezia globosa* CBS 7966, *Malassezia furfur* CBS 14141, *Malassezia sympodialis* CBS 7222, *S. epidermidis* KCTC 13172, and *S. aureus* NCTC 8325-4 were purchased from the Korean Collection for Type Cultures (KCTC, Korea). *Malassezia* species were cultured on Leeming and Notman agar (LNA; 0.8% bile salt, 0.5% glucose, 0.1% glycerol, 1% peptone, 0.01% yeast extract, 0.05% glycerol monostearate, 1.3% agar, 0.05% Tween 60, and 0.5% whole fat cow milk, with pH adjusted to 6.0 using HCl) or in modified Dixon medium (mDixon; 3.6% malt extract, 0.6% peptone, 2% bile salt, 1% Tween 40, 0.2% glycerol, and 0.2% oleic acid) (30, 31). *Staphylococcus* species were cultured in tryptic soy broth (TSB; 1.7% pancreatic digest of casein, 0.3% papaic digest of soybean, 0.25% glucose, 0.5% NaCl, and 0.25% dipotassium phosphate) or mDixon medium (32).

### Culture conditions

*Malassezia* species were initially cultured on LNA at 34°C for 3–4 days. Then, the cells were transferred to a 250 mL baffled flask containing 50 mL of mDixon medium and incubated at 34°C with shaking at 160 rpm for 24 h. Subsequently, the OD_600_ of the culture in mDixon medium was adjusted to 1.0 before co-cultivation or acid treatment. For co-cultivation, *Staphylococcus* species were first grown on TSA at 37°C for 1 day. Then, the bacteria were transferred to TSB medium in a 14 mL round-bottom tube and incubated at 37°C with shaking for 24 h. Next, the cultured bacteria were inoculated into *Malassezia* culture flasks at an OD_600_-based ratio of 0.1 or 0.01. For acid treatment, the pH of the medium was adjusted to 5.0 using HCl, lactic acid, or AcOH. Following incubation under these conditions for 12 h, the samples were used for Hi-C analysis, western blotting, ChIP-seq analysis, or RNA isolation.

### *In situ* Hi-C analysis of *Malassezia* species

Hi-C sequencing was performed according to a previously described protocol (18, 33). Briefly, *Malassezia* cells were fixed with paraformaldehyde for 10 min at 34°C to a final concentration of 1%; this was followed by 15 min of quenching using glycine. Next, the fixed *Malassezia* cells were harvested; in the case of co-cultured samples, *Malassezia* and *Staphylococcus* species were separated using a filter before being collected. The collected cells were bead-beaten using glass beads and then treated with SDS for nuclei permeabilization. DNA within the permeabilized nuclei was cleaved using *Mbo*I to generate 5′ overhangs, which were filled with biotinylated nucleotides and subsequently ligated *in situ*. Next, the DNA was sheared using a Bioruptor Pico sonicator (Diagenode, Belgium), and biotinylated ligation junctions were captured using streptavidin beads. The captured DNA was processed for library construction using the Illumina NEBNext Ultra II DNA Library Prep Kit (NEB, E7770). Finally, the libraries were submitted to Macrogen Inc. (Seoul, Korea) for paired-end (100 bp × 2) sequencing on a Novaseq6000 device (Illumina, Inc., San Diego, CA, USA). Hi-C data analysis is described in the Supplemental methods.

### Western blotting

Total protein was extracted by resuspending cells in lysis buffer and disrupting them via bead-beating with glass beads. Protein concentration was determined using a Qubit 4 fluorometer (ThermoFisher, Q33226), and either 25 or 50 µg of total protein was used. For western blot analysis, anti-H3 (Abcam, ab1791, 1:1,000 dilution), anti-H3K9ac (Abcam, ab177177, 1:2,500 dilution), or anti-H3K27ac (Abcam, ab4729, 1:5,000 dilution) primary antibodies, and anti-rabbit IgG (Bio-Rad, 1706515, 1:10,000 dilution) secondary antibodies, were used.

### ChIP-seq analysis

ChIP was performed according to a previously published protocol with modifications (18). Dyna-G beads (ThermoFisher, 10004D) were pre-incubated at 4°C with either anti-H3K9ac (Abcam, ab177177) or anti-H3K27ac (Abcam, ab4729) antibodies. Cultured cells were fixed with 3% paraformaldehyde at 18°C for 30 min, and then secondarily fixed with dimethyl adipimidate dihydrochloride (DMA) solution in PBS for 45 min at room temperature. The fixed cells were lysed in a buffer containing HEPES-KOH, NaCl, EDTA, Triton X-100, and phenylmethylsulfonyl fluoride, disrupted with glass beads, and sonicated for DNA shearing. The lysate was pre-cleared with lysis buffer-washed beads, and immunoprecipitation was conducted overnight at 4°C with antibody-conjugated beads. The beads were sequentially washed with lysis buffer, high-salt buffer, wash buffer, and TE buffer. Then, bound DNA was eluted, reverse-crosslinked overnight at 65°C, treated with proteinase K, and purified using the QIAquick PCR Purification Kit (Qiagen, 28104). The purified DNA was used for library preparation using the NEBNext Ultra II DNA Library Prep Kit (NEB, E7770), incorporating *S. cerevisiae* spike-in DNA at a 0.1 ratio relative to the quantity of input DNA. The libraries were sequenced using the method described above. Raw data were processed and mapped to the reference genome using the pipeline applied for Hi-C data. Mean reads per million (RPM) values were calculated for each gene, and heat maps, as well as anchor plots, were generated using EaSeq (34).

### RNA isolation and RNA-seq analysis

Total RNA was extracted through cell wall disruption via bead-beating using glass beads, and subsequently purified using the RNeasy Mini Kit (Qiagen, 74104) following the instructions of the manufacturer. Before further processing, residual DNase was removed from the purified RNA using a TURBO DNA-free kit (Invitrogen, AM1907). The concentration of the extracted RNA was determined using a Qubit fluorometer (ThermoFisher, Q33226). In total, 1 µg of the isolated RNA with 0.1 µg of *S. cerevisiae* spike-in RNA was used per library for mRNA isolation using the NEBNext Poly(A) mRNA Magnetic Isolation Module (NEB, E7490) following the protocol of the manufacturer; then, library preparation was carried out using the NEBNext Ultra II RNA Library Prep Kit for Illumina (NEB, E7770) according to the protocol of the manufacturer. The libraries were sequenced using the method described above. Sequencing adapter removal and quality-based trimming of raw reads were performed using Trimmomatic v0.36 (35). The cleaned reads were aligned to the reference genome using Bowtie2 (36) with default parameters. Read counts mapped to each coding sequence were obtained using featureCounts (37). The resulting count data were then normalized, and differentially expressed genes were identified using the DESeq2 (38) package for downstream analysis. GO term analysis was performed using BiNGO in Cytoscape (version 3.10.3).

## RESULTS

### Chromosomal interactions and nuclear organization in the *M. restricta* genome

**Fig 1:**
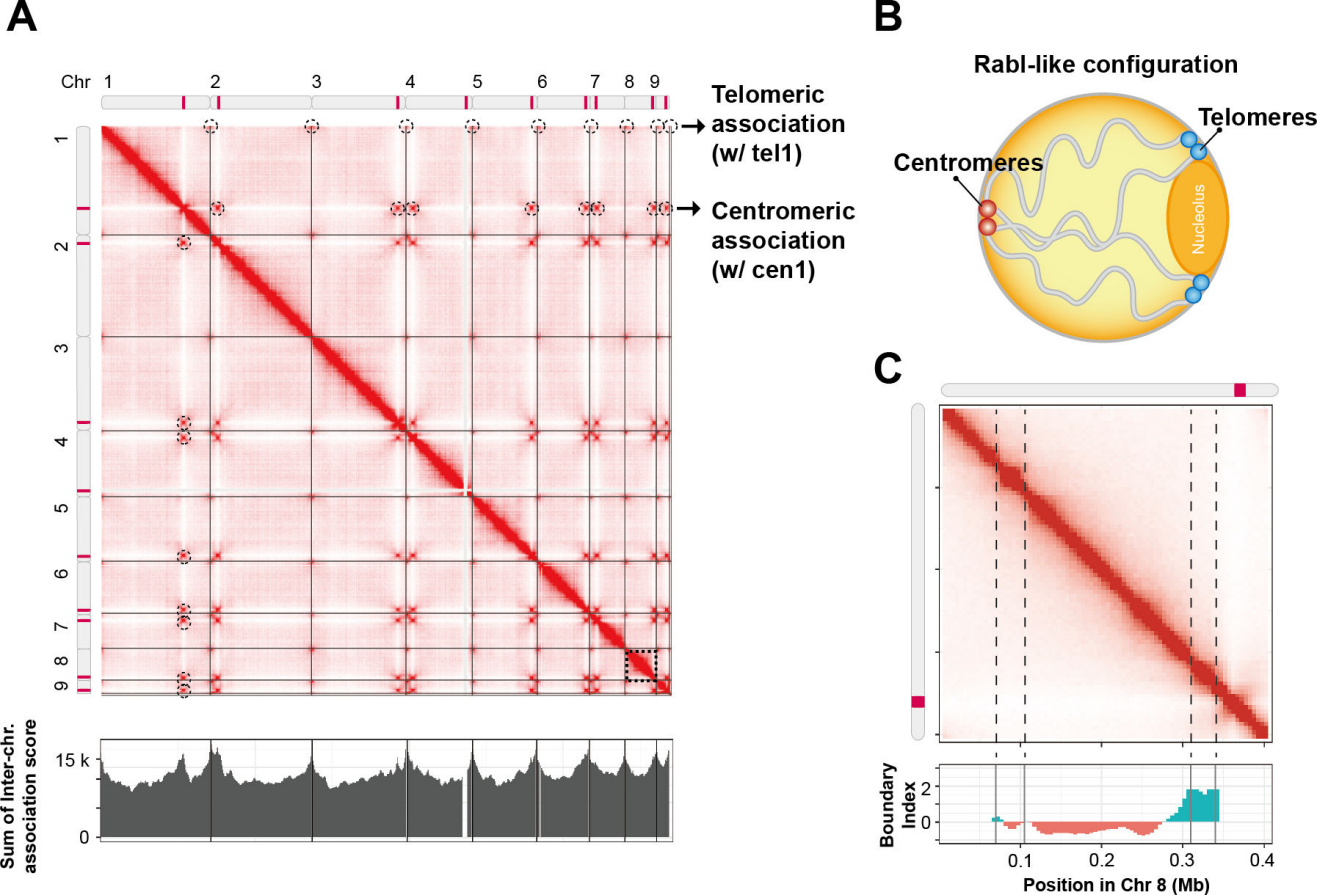
Chromosomal interactions and spatial organization in the *M. restricta* genome. (**A**) Hi-C contact map of the *M. restricta* genome. The map represents chromatin interaction frequencies across all nine chromosomes (Chr 1–9). The intensity of the red color indicates interaction strength, with darker shades denoting stronger interactions. Notable interaction hotspots include telomeric (*tel*) and centromeric (*cen*) regions (black arrows). The histogram represents the sum of inter-chromosomal association scores, highlighting regions of frequent interactions. (**B**) Schematic model of the Rabl-like chromosomal configuration in the nucleus. Centromeres (red) cluster at one side of the nucleus, while telomeres (blue) are localized toward the nuclear periphery. This arrangement resembles the classic Rabl-like configuration observed in various eukaryotic cells. (**C**) Chromatin interaction landscape of Chromosome 8. The Hi-C contact map (top) reveals structured interaction domains. The boundary index (bottom) marks potential TAD boundaries.

The Hi-C contact map revealed significant intrachromosomal interactions along the diagonal, consistent with the expected spatial proximity of adjacent genomic regions. Additionally, distinct inter-chromosomal interaction hotspots were observed, particularly in the centromeric and telomeric regions, suggesting a spatial organization in which centromeres and telomeres formed clusters within the nuclear space (Fig. 1A). Centromeric interactions were detected as inter-chromosomal contact hotspots, indicative of centromeric clustering, a phenomenon widely observed in fungal and yeast genomes. Similarly, telomeric associations were identified, suggesting telomeric clustering at the nuclear periphery.

Centromeres serve as key structural elements in chromosomal organization; however, their precise positions remain undefined in many non-model organisms. Previous studies have demonstrated that centromeres tend to cluster within the nucleus, forming strong inter-chromosomal interactions (17). To infer putative centromeric positions in *M. restricta*, we leveraged Hi-C inter-chromosomal association scores (Fig. 1; Fig. S1). By summing the inter-chromosomal association scores along each chromosome, we identified distinct peaks indicative of highly interacting centromeric regions (Fig. S1). A summary of the predicted centromeric positions, binned at 2 Kb intervals, is provided in Table S1. To identify the location of centromeres with precision, we analyzed the GC ratio in regions with the highest inter-chromosomal associations, scanning every 500 bp. We identified a region with GC content ranging from 25% to 31%, which is significantly lower than the average GC content (55.7%). In a previous study, Sankaranarayanan et al. predicted the centromeric positions of *M. restricta* CBS877 based on its low GC content (39). However, we identified several inaccuracies in their predictions, such as chromosome misidentification and inversions, which we have corrected. To define the centromere positions of *M. restricta*, we used the genome of strain KCTC 27527, which contains consistent telomeric repeat motifs (CACTMA-TKAGTG) at both ends of all chromosomes. This canonical feature enabled clear determination of chromosomal orientation and accurate centromere mapping. In contrast, the CBS 7877 genome used by Sankaranarayanan et al. shows inconsistencies, most notably, chromosomes I and VII appear in reverse complement orientation, and chromosome V lacks a predicted centromere. Our analysis, based on the structurally consistent KCTC 27527 genome, provides a more reliable and biologically coherent centromere map. Of note, our centromeric predictions were closely aligned with the corrected GC-based data (39), reinforcing the theory that centromeres exhibit the strongest inter-chromosomal associations. These findings suggest that centromeric positions can be reliably predicted using inter-chromosomal association scores.

To better understand the spatial organization of *M. restricta* chromosomes, we established a nuclear arrangement model with a Rabl-like configuration. This model showed that centromeres cluster near one side of the nucleus, while telomeres are localized toward the nuclear periphery (Fig. 1B). This organization typifies the chromosomal arrangement observed in various eukaryotic species, where centromeric clustering facilitates chromosome movement during mitosis and telomere anchorage to the nuclear envelope contributes to genome stability and transcriptional regulation.

Beyond centromeric and telomeric organization, we sought to characterize higher-order chromatin structures by identifying TADs in *M. restricta*. Using the boundary index, we identified 69 TAD boundaries under the *M. restricta* axenic culture, indicative of well-defined chromatin domains (Fig. 1C; Fig. S2). The boundary index indicates regions of chromatin compartmentalization, providing insights into the hierarchical organization of chromatin interactions. These findings establish a reference framework for TAD formation in *M. restricta*, paving the way for further investigations into the influence of environmental factors on chromatin domain stability and nuclear architecture.

### Co-culture with *S. epidermidis* induces chromatin remodeling in *M. restricta*

**Fig 2:**
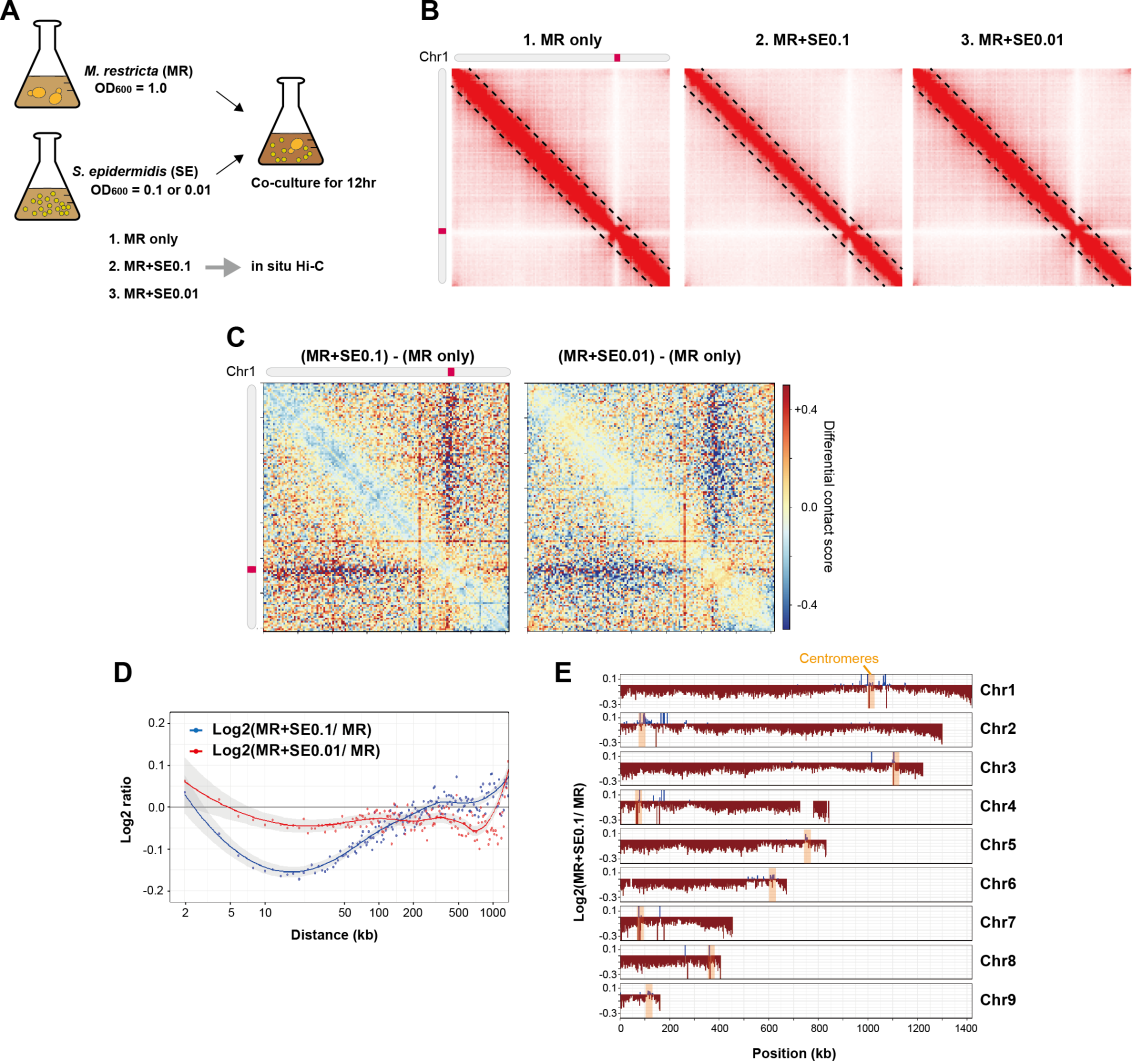
Chromatin reorganization in *M. restricta* under *S. epidermidis* co-culture. (**A**) Experimental design of the *in situ* Hi-C analysis. *M. restricta* (MR) was cultured alone or co-cultured with *S. epidermidis* (SE) at two initial optical densities (OD_600_ = 0.1 and 0.01) for 12 h. Three conditions were analyzed: 1. MR only, 2. MR+SE.1, and 3. MR+SE.01. (**B**) Hi-C contact maps for chromosome 1 under different experimental conditions. Maps depicting chromatin interaction frequencies under the MR only, MR+SE.1, and MR+SE.01 conditions. Black dashed lines indicate interaction boundaries at a 50 Kb distance. (**C**) Differential contact maps comparing co-culture conditions (MR+SE) to the MR-only condition. Hi-C log_2_ ratio maps showing chromatin interaction differences. (**D**) Genome-wide changes in contact probability as a function of genomic distance. Log_2_ ratio of interaction frequencies between the MR+SE and MR only conditions plotted against genomic distance. (**E**) Chromosome-wide effects of *S. epidermidis* co-culture. Local interactions within a length of 100 Kb were extracted, and the log_2_ ratio of interaction scores (MR+SE.1/MR) was computed. Average values for each 2 Kb bin were plotted. While most genomic regions exhibited a decrease in interaction frequency (brown), centromeric regions (orange) displayed a relative increase.

To investigate the mechanisms by which microbial interactions influence chromatin architecture, we co-cultured *M. restricta* (MR) and *S. epidermidis* (SE) at different initial ratios (Fig. 2A). Given that *S. epidermidis* multiplies at a faster rate than *M. restricta*, we adjusted the starting inoculum by mixing *M. restricta* cells with 10-fold (MR+SE.1) or 100-fold (MR+SE.01) fewer *S. epidermidis* cells to maintain a balanced co-culture environment over 12 h. Following incubation, we performed *in situ* Hi-C analyses to assess genome-wide chromatin interactions in response to the co-culture conditions.

Hi-C contact maps revealed that *M. restricta* maintained a stable global chromatin organization during coculture (Fig. 2B). However, under the MR+SE.1 condition, localized alterations in chromatin interactions were observed, particularly at genomic distances less than 50 Kb. In contrast, no detectable changes were observed under the MR+SE.01 condition (Fig. 2B). These findings suggest that co-culture with *S. epidermidis*, particularly at a high bacterial density, can subtly but significantly affect fine-scale chromatin architecture in *M. restricta*.

To determine whether co-culture conditions affect higher-order chromatin organization, we compared TAD boundary structures between the MR and MR+SE.1 conditions using the boundary index (Fig. S2). Under the MR axenic and MR+SE.1 culture, we detected 69 and 74 TAD boundaries, respectively. Despite this minor variation, overall TAD number and positioning remained mostly conserved, suggesting that chromatin compartmentalization in *M. restricta* is stable even in the presence of *S. epidermidis*.

To further evaluate the effect of *S. epidermidis* co-culture on chromatin interactions, we generated differential contact maps comparing co-culture conditions with MR axenic culture (Fig. 2C). Of note, as compared to the MR+SE.01 condition, the MR+SE.1 condition induced more significant changes in chromatin interactions, characterized by a decrease in short-range interactions and a localized increase in long-range interactions (Fig. 2C). These changes suggest a chromatin remodeling response to *S. epidermidis* co-culture, with a greater effect observed with higher *S. epidermidis* density (MR+SE.1). Chromosome-specific analyses confirmed an extensive decrease in short-range interactions across most chromosomes under the MR+SE.1 condition. In contrast, the MR+SE.01 condition induced weaker and more localized changes (Fig. 2C; Fig. S3), suggesting that the effect of bacterial co-culture on chromatin organization is dose-dependent.

Genome-wide contact probability analyses showed that *S. epidermidis* co-culture modulated chromatin interactions in a distance-dependent manner (Fig. 2D). Short-range interactions (5–100 kb) decreased under the MR+SE.1 condition, whereas long-range interactions (>500 kb) increased under the MR condition. A similar but weaker effect was observed under the MR+SE.01 condition (Fig. 2D), highlighting the dose-dependent relationship between *S. epidermidis* abundance and chromatin changes in *M. restricta*.

Further analysis of changes in chromatin interaction revealed a general decrease in chromatin compaction across most genomic regions (Fig. 2E). This suggests that microbial co-culture induced chromatin architecture relaxation, which may have facilitated transcriptional reprogramming. In contrast to the global decrease in chromatin interactions, centromeric regions exhibited increased interaction frequency (Fig. 2E, orange highlights). These findings indicate that *S. epidermidis* co-culture exerts a dual effect on chromatin architecture, i.e., a genome-wide decrease in chromatin interactions, accompanied by localized centromeric clustering. This remodeling effect suggests that microbial interactions trigger a coordinated chromatin reorganization process, which has potential implications for transcriptional regulation and cellular adaptation.

### Co-culture with *S. aureus* does not induce chromatin remodeling in *M. restricta*

To determine whether the chromatin remodeling effect observed in *M. restricta* was specific to *S. epidermidis*, we performed Hi-C analyses on MR co-cultured with *S. aureus* (MR+SA.1) (Fig. S4A and B). In contrast to the extensive chromatin changes observed under the MR+SE.1 condition, the genome-wide Hi-C interaction matrix under the MR+SA.1 condition remained highly similar to that observed under the MR-only condition. Differential contact maps across all chromosomes revealed no significant changes in chromatin interactions in two biological replicates (Fig. S4C and D), indicating that *S. aureus* co-culture did not induce major chromatin remodeling in *M. restricta*.

These findings suggest that the chromatin reorganization observed under *S. epidermidis* co-culture conditions is a species-specific response rather than a general consequence of microbial co-culture. The distinct alteration of short-range chromatin contacts by *S. epidermidis* suggests a unique interkingdom interaction mechanism potentially mediated by environmental stress, metabolite exchange, or interspecies signaling pathways.

### Acetic acid induces chromatin reorganization in *M. restricta*

**Fig 3:**
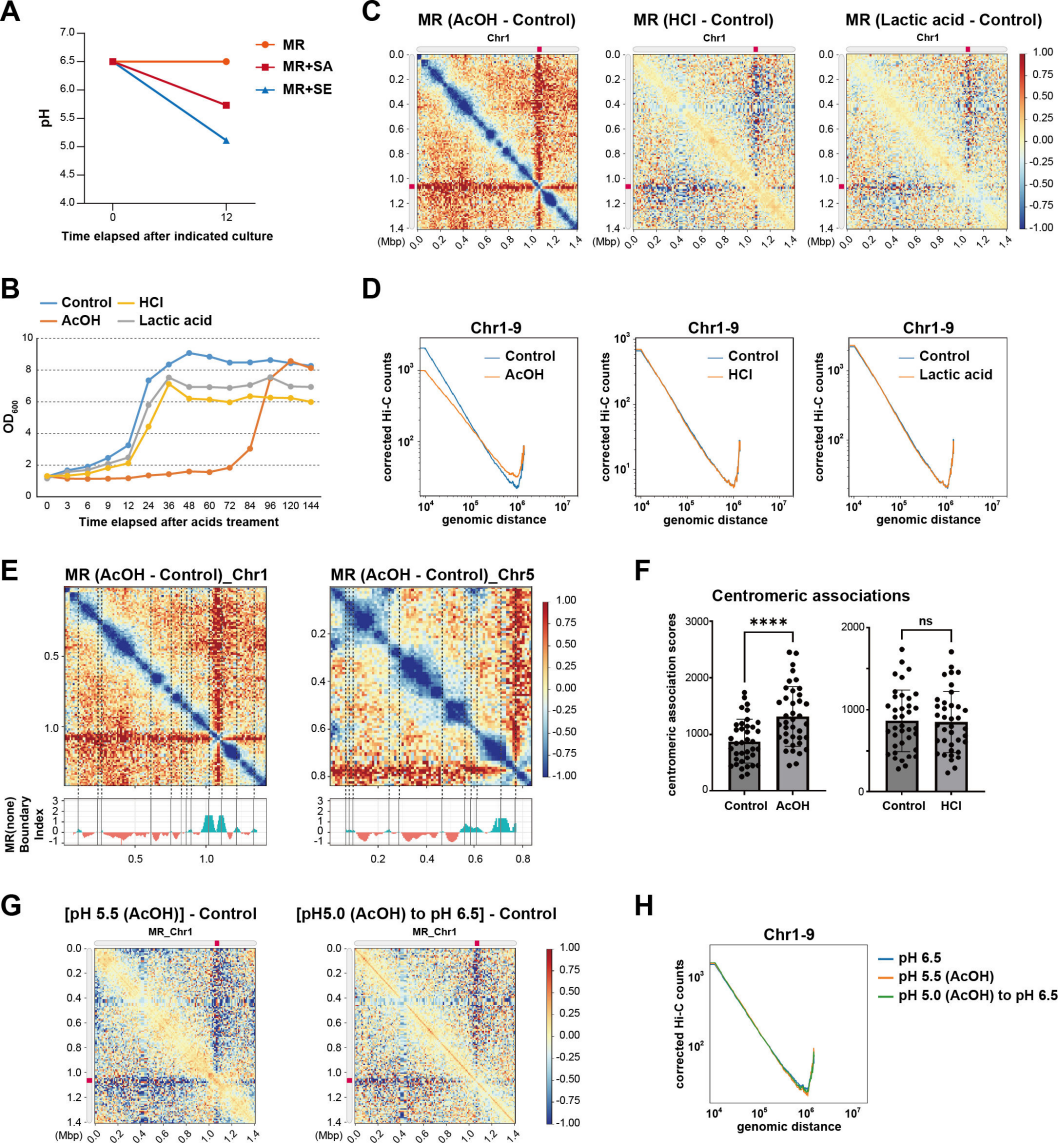
Acetic acid induces chromatin reorganization in *M. restricta.* (**A**) pH measurements under different culture conditions. The pH of the media was measured under MR axenic culture, co-culture with *S. aureus* (MR+SA.1), and co-culture with *S. epidermidis* (MR+SE.1) for 12 h. (**B**) Growth curve of *M. restricta* under acid stress. The pH of the mDixon medium was adjusted to 5.0 using AcOH, HCl, or lactic acid, and this medium was used for *M. restricta* culturing. Optical density (OD_600_) was measured at the indicated time points (up to 144 h) to assess cell growth under each condition. (**C**) Differential chromatin contact maps of *M. restricta* under acid treatment. Hi-C log_2_ ratio maps for Chr1 under the AcOH (left), HCl (middle), and lactic acid (right) treatment conditions. (**D**) Genome-wide chromatin contact probability curves averaged across chromosomes 1–9, comparing each acidic treatment (AcOH, HCl, lactic acid) to the control. Contact probability curves averaged across chromosomes 1–9 were plotted against genomic distance. (**E**) Changes in local chromatin interaction following AcOH treatment. Hi-C differential contact maps for Chr1 (left) and Chr5 (right) reveal disruptions in TAD formation. Boundary index plots (bottom) generated from axenic culture samples (Fig. S2) indicate reference TAD boundary positions. (**F**) AcOH enhances centromeric clustering. Quantification showed a significant increase in centromere–centromere interaction frequencies (****: *P* < 0.0001) under AcOH treatment; however, no significant change was observed under HCl treatment. (**G**) Differential contact maps comparing milder acidic conditions: pH 5.5 AcOH treatment (left) and AcOH treatment at pH 5.0 subsequently adjusted to pH 6.5 with NaOH (right) to control conditions across chromosomes 1 in *M. restricta*. (**H**) Genome-wide chromatin contact probability curves corresponding to conditions in panel G.

Since co-culture with *S. epidermidis* significantly lowered the pH of the medium to approximately 5.0 and co-culture with *S. aureus* resulted in a pH of approximately 5.7 (Fig. 3A), we sought to determine whether acidic stress alone could account for the chromatin remodeling observed. Thus, we exposed *M. restricta* to acetic acid (AcOH), hydrochloric acid (HCl), and lactic acid, adjusting the media pH to 5.0 to mimic the acidic conditions of the co-culture system (10). Then, we assessed their effects on fungal growth and chromatin organization. Growth curve analyses revealed that AcOH significantly inhibited *M. restricta* growth, while HCl and lactic acid exerted milder effects (Fig. 3B), suggesting that AcOH induces physiological stress distinct from that induced by general pH reduction.

Hi-C analyses revealed that treatment with AcOH induced extensive chromatin reorganization, while treatment with HCl and lactic acid induced minimal effects on global chromatin architecture (Fig. 3C). AcOH treatment induced extensive loss of chromatin interactions, particularly in short-range contacts, indicating chromatin decompaction (Fig. 3D; Fig. S5). Of note, this pattern closely resembled the chromatin changes observed under *S. epidermidis* co-culture (MR+SE.1), suggesting that AcOH is a key driving factor for chromatin remodeling. Distance-based chromatin interaction analyses showed that treatment with AcOH induced a genome-wide decrease in proximal genomic associations, particularly at distances <10^5^ Kb. In contrast, treatment with HCl and lactic acid exerted no significant effects on the frequency of genomic associations (Fig. 3D; Fig. S5).

Notably, the loss of chromatin interactions significantly overlapped with TAD boundaries, suggesting that AcOH preferentially disrupted chromatin association within these domains (Fig. 3E). This finding indicates that AcOH-induced chromatin remodeling is not a stochastic process but one that specifically affects TAD integrity, leading to large-scale alterations in chromatin compartmentalization and structural organization.

In contrast to the general decrease in chromatin interactions observed, centromeric interactions were significantly enhanced under AcOH treatment (Fig. 3F). This suggests that while local chromatin domains undergo decompaction, centromeres exhibit increased clustering, potentially serving as a structural adaptation to acidic stress. However, HCl treatment did not significantly affect centromeric associations (Fig. 3F), further supporting the notion that the chromatin remodeling effects observed are specific to AcOH rather than being a general response to pH reduction.

To investigate whether the decrease in local chromatin interactions caused by AcOH treatment is due to the chemical properties of AcOH alone or a combined effect with lower pH, we examined the impact of medium conditions adjusted to pH 5.5 (a milder acidic condition) and pH 6.5 (adjusted with NaOH after AcOH treatment). In both conditions, we did not observe a reduction in local chromatin interactions compared to the pH 6.5 control condition (Fig. 3G and H; Fig. S6). These results suggest that a pH of 5.0, combined with AcOH treatment, is required to induce the observed chromatin decompaction and loss of local associations. This indicates that the chromatin reorganization observed under AcOH treatment is primarily driven by both the chemical properties of AcOH and the lower pH, rather than solely by the AcOH treatment itself.

Thus, our findings demonstrate that AcOH, in the context of acidic stress (pH 5.0), acts as a key factor in chromatin remodeling in *M. restricta*, leading to a coordinated nuclear reorganization process, including the weakening of local chromatin interactions and the increased clustering of centromeres. These results further highlight how microbial-derived metabolites, such as AcOH, influence chromatin architecture and gene expression, contributing to the response to environmental stress in the skin microbiome.

### Acetic acid induces conserved chromatin remodeling in *M. sympodialis*

**Fig 4:**
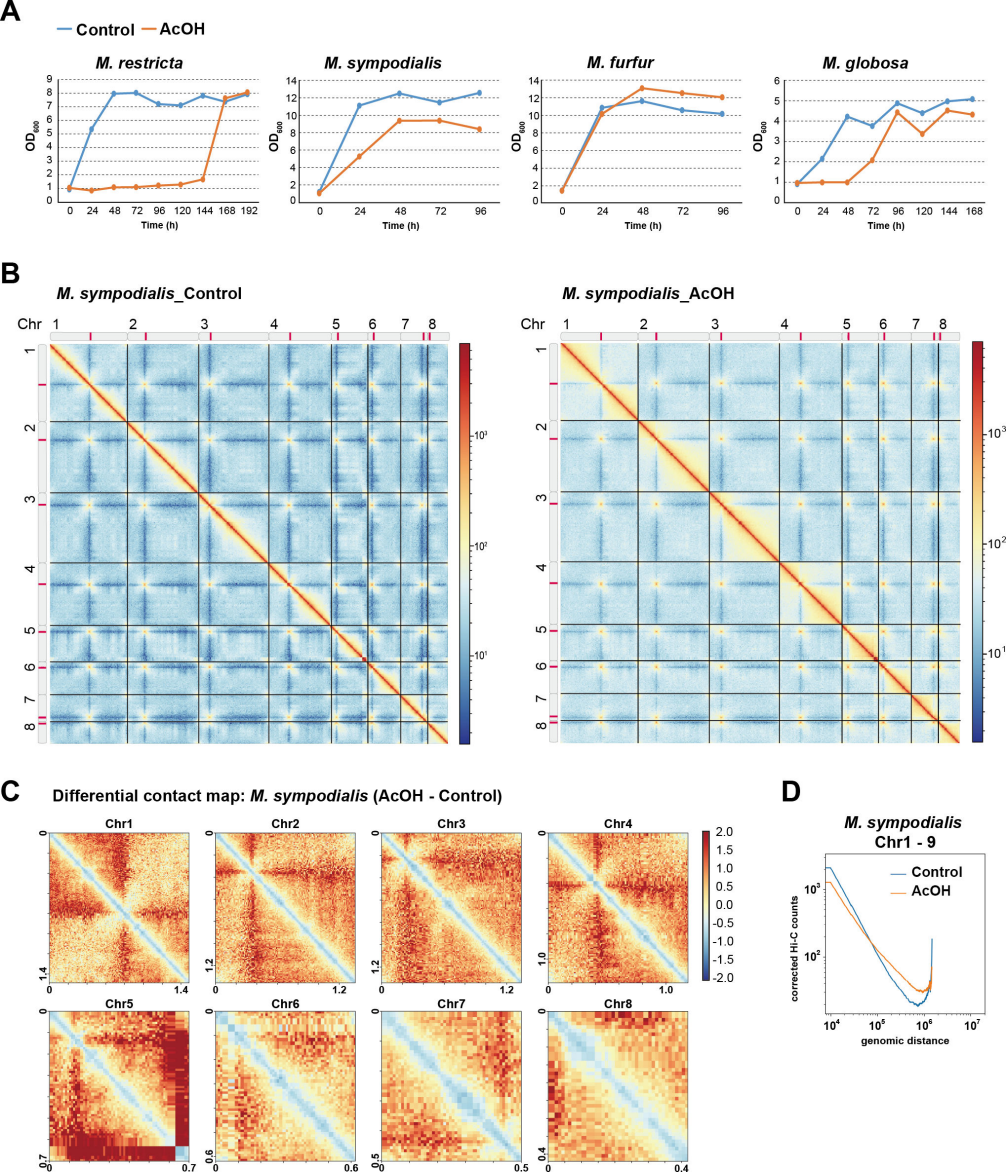
*M. sympodialis* chromatin response to acetic acid stress. (**A**) Growth curves for *Malassezia* species under AcOH treatment. OD_600_ values for *M. restricta, M. sympodialis, M. furfur,* and *M. globosa* were determined under the control (blue) and AcOH treatment (red) conditions. (**B**) Hi-C contact maps for all *M. sympodialis* chromosomes under the control (left panel) and AcOH treatment conditions (right panel). (**C**) Differential contact maps for *M. sympodialis* chromosomes following AcOH stress. Hi-C log_2_ ratio maps showing differences in chromatin interaction. Increased and decreased interactions under co-culture conditions are indicated in red and blue, respectively. (**D**) Genome-wide chromatin contact probability curves. Contact probability curves averaged across chromosomes 1–9 are plotted against genomic distance.

As *M. restricta* exhibited severe growth defects under AcOH treatment (Fig. 3B), we sought to determine whether AcOH-induced chromatin remodeling was a direct consequence of growth inhibition or a distinct effect of the acid. To clarify these effects, we evaluated chromatin response to AcOH in *M. sympodialis*, a *Malassezia* species closely related to *M. restricta* that maintains moderate growth in AcOH; this allowed us to assess chromatin alterations independently of severe growth suppression (Fig. 4A).

To characterize the three-dimensional genome organization of *M. sympodialis*, we generated Hi-C contact maps under controlled conditions (Fig. 4B, left). The *M. sympodialis* genome comprises eight chromosomes, and its Hi-C interaction matrix revealed strong intra-chromosomal interactions along the diagonal, indicating well-organized chromatin compartmentalization. Additionally, we observed inter-chromosomal centromeric and telomeric associations similar to those observed in the *M. restricta* Hi-C interaction matrix (Fig. 1A and 4B). Under AcOH treatment, overall chromatin structure remained intact (Fig. 4B, right), suggesting that global chromatin organization was largely preserved despite acid exposure. However, differential contact maps showed localized chromatin interaction changes across all chromosomes. As with *M. restricta*, AcOH exposure significantly reduced local chromatin interactions, particularly in TAD-associated regions, in *M. sympodialis* (Fig. 4C and D). In contrast to the genome-wide decrease in local chromatin interactions, our Hi-C contact probability analysis revealed an increase in centromeric associations under AcOH stress (Fig. 4C and D). This mirrors the centromeric clustering response observed in *M. restricta*, suggesting that AcOH may induce a conserved nuclear reorganization pattern across *Malassezia* species. The simultaneous weakening of local chromatin interactions and strengthening of centromeric clustering suggests that AcOH-induced chromatin remodeling follows a common mechanism in these fungal species, possibly representing an adaptive nuclear response to environmental stress. However, further investigation across diverse clades is needed to determine whether this response is conserved throughout the genus.

### Acetic acid treatment induces redistribution of histone acetylation

**Fig 5:**
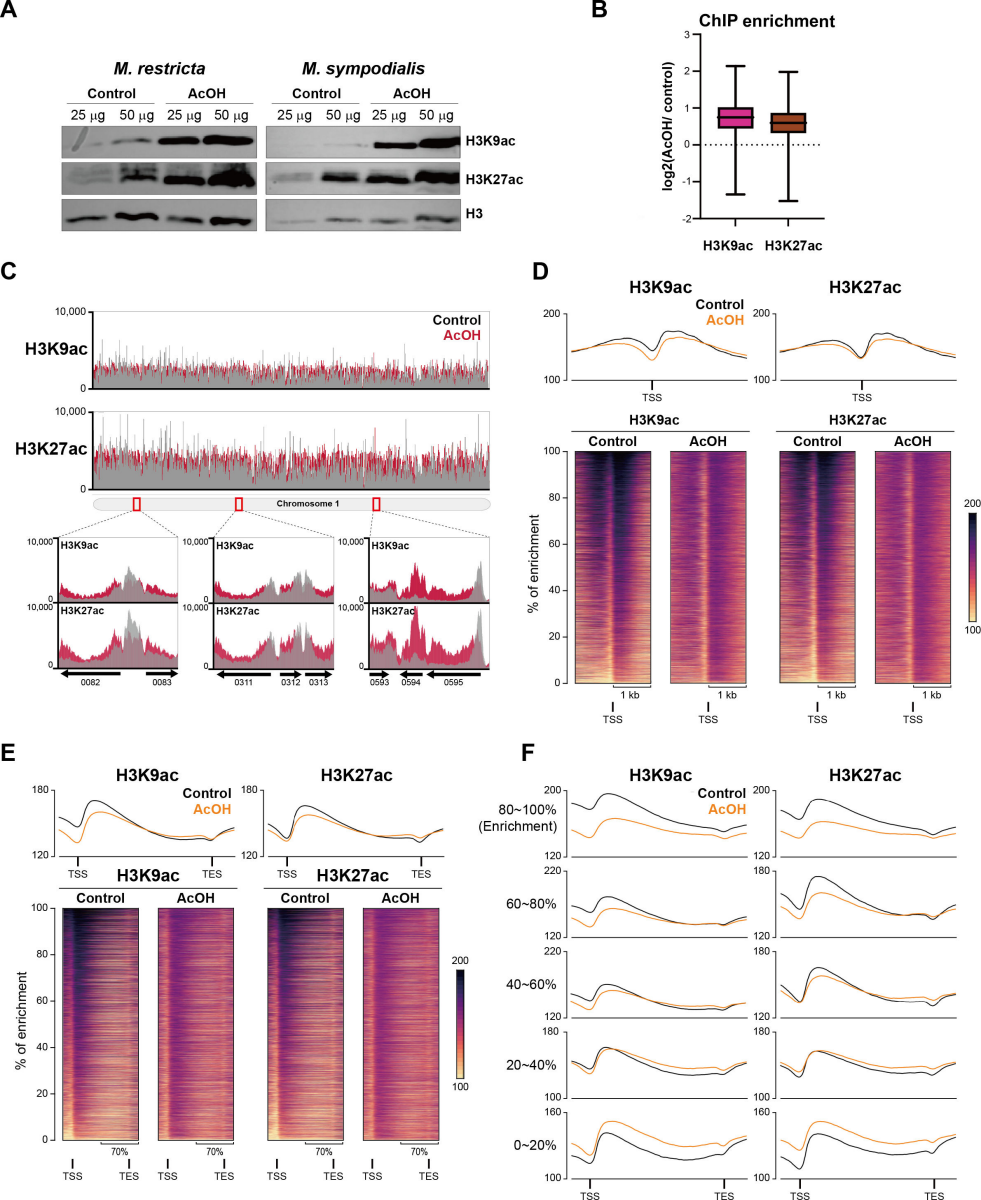
Acetic acid redistributes histone acetylation in *M. restricta.* (**A**) Western blot analysis of histone acetylation. H3K9ac and H3K27ac levels were assessed in *M. restricta* and *M. sympodialis* under the control and AcOH treatment conditions. (**B**) Box plot comparison of genome-wide H3K9ac and H3K27ac ChIP enrichment in *M. restricta*. (**C**) Genome-wide H3K9ac and H3K27ac ChIP-seq signal distribution in chromosome 1 of *M. restricta*. (**D**) Redistribution of histone acetylation near the transcription start site (TSS). Pileup plots (upper) and heatmaps (lower) showing decreased acetylation near TSS regions and increased acetylation extending into gene bodies. (**E**) Changes in histone acetylation across gene bodies from TSSs to transcription end sites (TESs). Highly acetylated regions exhibited decreased acetylation, while weakly acetylated regions exhibited increased acetylation. (**F**) Peak-based stratification of changes in histone acetylation. Highly acetylated peaks lost acetylation, while weakly acetylated peaks gained acetylation.

To investigate the effect of AcOH on chromatin organization, we evaluated histone acetylation levels in *M. restricta* and *M. sympodialis* through western blotting. Both species exhibited a significant increase in H3K9ac and H3K27ac protein levels following AcOH treatment (Fig. 5A), indicating that AcOH broadly alters histone acetylation patterns.

To distinguish the acetylation levels of nucleosome-incorporated histones from those of newly synthesized, pre-positioned histones, we conducted ChIP-seq analyses on H3K9ac and H3K27ac in *M. restricta*. To normalize total read counts across samples, *S. cerevisiae* spike-in DNA was added at a 1:10 ratio relative to the quantity of input DNA. Box plot analyses showed that AcOH treatment significantly increased overall histone acetylation ChIP-seq signals (Fig. 5B), indicating that H3K9ac and H3K27ac were highly expressed in nucleosomes rather than being excluded.

We observed significant redistribution of histone acetylation across the genome rather than a uniform increase. Under normal conditions, H3K9ac and H3K27ac are concentrated at transcription start sites (TSS). However, in AcOH-treated cells, these acetylation markers were more widely distributed across gene bodies instead of clustering at the promoter regions (Fig. 5C). More in-depth examination of TSS revealed a marked decrease in both H3K9ac and H3K27ac signals at the promoter regions (Fig. 5D). Heatmaps further demonstrated a shift in acetylation patterns, with decreased enrichment at TSS regions and a concurrent increase in acetylation along gene bodies (TSS-transcription end sites [TES]) (Fig. 5D and E). These findings suggest that AcOH does not simply elevate histone acetylation levels but redistributes them across transcriptional units, potentially altering regulatory dynamics. Stratification of acetylation peaks based on baseline enrichment levels revealed further redistribution patterns. Regions with high initial acetylation signals tended to lose acetylation upon AcOH treatment, whereas regions with low initial acetylation exhibited increased signals (Fig. 5E and F). This reciprocal pattern supports the idea of a global rebalancing of histone acetylation rather than a simple additive effect.

Collectively, these findings suggest that AcOH-induced loss of promoter-associated histone acetylation may contribute to the local loosening of chromatin structure, reinforcing a mechanistic link between epigenetic changes and altered 3D genome organization during acid stress.

### Transcriptomic consequences of AcOH and HCl treatment in *M. restricta*

To investigate the molecular consequences of AcOH-induced chromatin remodeling, we performed RNA-seq analysis on *M. restricta* treated with AcOH or HCl, with the pH of the medium adjusted to 5.0 in each case. This experimental design allowed us to distinguish AcOH-specific transcriptional effects from general acid stress responses. To normalize total RNA levels across samples, we added *S. cerevisiae* spike-in RNA at a 1:10 ratio relative to the quantity of total *M. restricta* RNA.

**Fig 6:**
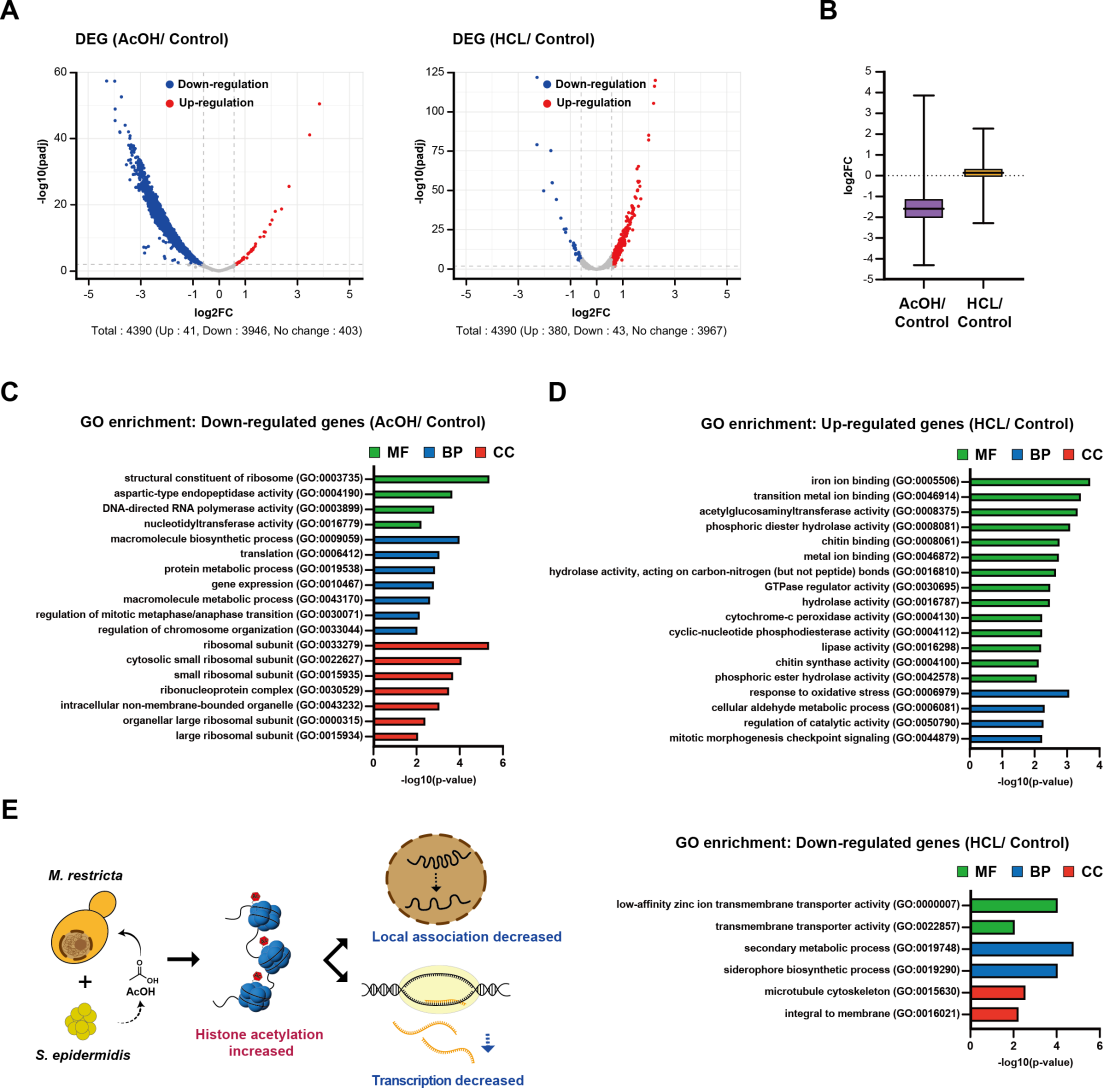
Transcriptomic responses of *M. restricta* to treatment with acetic acid and hydrochloric acid. (**A**) Volcano plots of differentially expressed genes (DEGs) in *M. restricta* treated with AcOH and HCl (pH 5.0). Genes with fold change (FC) ≥1.5 or ≤−1.5 were considered significantly up- or downregulated, respectively. (**B**) Box plot comparing FC in overall transcript levels between the AcOH and HCl treatments. (**C**) GO analysis of downregulated genes (FC ≤ −8, *n* = 75) upon treatment with AcOH (MF, molecular function; BP, biological process; CC, cellular component). (**D**) GO analysis of upregulated (FC ≥ 1.5, *n* = 381) and downregulated genes (FC ≤ −1.5, *n* = 43) upon treatment with HCl. (**E**) Schematic model summarizing chromatin and transcriptional changes in *M. restricta* under *S. epidermidis* co-culture. Chromatin decompaction correlated with transcriptional repression, suggesting an association between chromatin structure and gene regulation.

RNA-seq analyses revealed that AcOH treatment resulted in extensive transcriptional suppression, with 3,946 genes downregulated and only 41 genes upregulated (fold change [FC] ≥1.5 or ≤−1.5; Fig. 6A; Table S2 and S3). This global decrease in gene transcript levels was further supported by a significant decrease in average gene expression (Fig. 6B), indicating that AcOH exerts a strong repressive effect on gene transcription. Although the 41 upregulated genes (FC ≥ 1.5) showed no significant enrichment in any GO categories, GO analysis of the most strongly downregulated genes (FC ≤ –8; *n* = 75) identified major biological pathways disrupted by AcOH exposure (Fig. 6C). Genes involved in translation and ribosome biogenesis (GO:0003735, GO:0022627, and GO:0006412), macromolecule metabolism (GO:0043170 and GO:0006397), and chromosome organization (GO:0051276) were significantly downregulated, suggesting that AcOH exposure alters fundamental cellular processes. Of note, genes encoding aspartyl proteases (GO:0004190), such as MRET_3770 and MRET_3104, which are known virulence-associated factors in *Malassezia* species, were also significantly downregulated, indicating that AcOH-induced chromatin remodeling may affect cellular adaptation and pathogenicity (Fig. 6C).

In contrast to the widespread repression of gene expression observed under AcOH treatment, exposure to HCl elicited a more limited transcriptomic response, with only 380 and 43 genes upregulated and downregulated, respectively (FC ≥1.5 or ≤−1.5; Fig. 6A; Table S4 and S5). Notably, only 5 out of the 43 genes downregulated by HCl treatment (FC ≤ –1.5) overlapped with the 75 genes downregulated by AcOH exposure (FC ≤ –8), indicating that AcOH and HCl treatments affect gene expression through distinct mechanisms rather than via a common low pH effect. GO term analysis of genes upregulated under HCl exposure revealed enrichment of iron-binding and transition metal-related proteins (GO: 0005506, GO: 0046872, and GO: 0046914; Fig. 6D), suggesting that *M. restricta* modulates iron and transition metal availability under HCl stress. Additionally, we observed increased expression of genes related to hydrolase activity (GO: 0016787, GO: 0016298), possibly reflecting a stress-induced response to facilitate nutrient acquisition or cell wall remodeling. Genes associated with oxidative stress responses (GO: 0006979) were also upregulated, indicating metabolic adjustments in response to acid stress. Conversely, siderophore biosynthetic process-associated biomolecules (GO: 0019290), such as ferricrocin synthase, were significantly downregulated, suggesting a lesser need for active iron acquisition. Given that siderophores are typically produced for iron scavenging under limiting conditions, their suppression implies that iron may become more freely available in acidic environments, possibly because of the altered solubility linked to pK value shifts. Additionally, the downregulation of membrane transport-related genes (GO: 0022857, GO: 0016021) suggests changes in nutrient uptake mechanisms and membrane composition, possibly reflecting adaptive restructuring in response to acid stress (Fig. 6D).

These findings demonstrate that AcOH treatment induces a major transcriptomic shift characterized by extensive gene repression, particularly of genes involved in translation-, metabolism-, and virulence-associated pathways. In contrast, HCl elicits a more targeted stress response, primarily affecting iron homeostasis and hydrolase activity. The correlation between AcOH-induced chromatin remodeling and global transcriptional suppression suggests that chromatin structure plays a central role in regulating gene expression under AcOH-induced environmental stress conditions.

## DISCUSSION

This study provides new insights into the mechanism by which microbial metabolites modulate chromatin architecture and gene expression in the skin microbiome. We demonstrate that *S. epidermidis*, a dominant skin commensal, induces extensive chromatin remodeling in *M. restricta* through AcOH secretion. Using an integrative multi-omics approach combining Hi-C, ChIP-seq, and RNA-seq, we showed that AcOH acts as an interkingdom epigenetic modulator, redistributing histone acetylation, inducing chromatin decompaction, and globally repressing transcription (Fig. 6). These findings align with those of previous studies, which showed that AcOH metabolism yields acetyl-CoA, which not only increases histone acetylation by enhancing substrate availability but also inhibits histone deacetylase activity (29, 40, 41). Histone acetylation neutralizes the positive charge of lysine residues, reducing charge-dependent interactions with nucleosomal or linker DNA, as well as interactions with neighboring histones, making DNA more accessible to the transcription machinery (42). The elevated histone acetylation levels observed under AcOH treatment possibly drive significant chromatin decompaction, altering 3D genomic organization. This structural reorganization may, in turn, enable transcriptional reprogramming and modulate gene expression patterns. Despite the overall decrease observed in chromatin interactions, inter-centromeric clustering significantly increased under AcOH treatment (Fig. 3F), suggesting that centromeres may serve as structural anchors during environmental stress. Although centromeric clustering has been well characterized in *S. cerevisiae* and *S. pombe* [3, 5, 6, 35], its functional role in *Malassezia* species under acidic conditions represents a novel finding.

ChIP-seq analyses demonstrated a global increase in histone acetylation under AcOH stress; however, this enrichment was redistributed from promoter regions to gene bodies (Fig. 5). Although acetylation typically promotes gene activation, this shift coincided with extensive gene repression, particularly of genes involved in translation-, metabolism-, and virulence-related pathways. These changes may reflect an adaptive response mediated by acetyl-CoA-dependent histone acetyltransferases, enabling *M. restricta* to adjust its transcriptional program under AcOH stress.

The chromatin alterations observed following AcOH treatment were more significant than those observed under *S. epidermidis* co-culture, possibly due to differences in AcOH concentration and exposure time. Under co-culture conditions, AcOH gradually accumulates, reaching approximately 25 mM after 24 h (10); however, under the direct treatment condition, approximately 20 mM AcOH was added at the beginning to achieve a pH of 5.0, resulting in immediate and sustained exposure. In consonance with this finding, milder AcOH treatment, with pH adjusted to 5.5, induced minimal chromatin changes, suggesting that a threshold concentration is required to induce structural changes. The dissociation state of AcOH and its membrane permeability vary with environmental pH, as described by the Henderson-Hasselbalch equation. Upon acidification toward its pKa of 4.75, a greater proportion of AcOH exists in the undissociated form, which can more readily diffuse across the cell membrane. For instance, at pH 5.0, approximately 36.5% of AcOH remains undissociated, compared to about 15.4% at pH 5.5, a more than twofold difference. This increased membrane permeability likely contributes to the chromatin remodeling observed under pH 5.0 conditions (Fig. 3C and G). Notably, our co-culture experiments showed that the medium pH dropped to ~5.0 after 12 h of incubation with *S. epidermidis* and *M. restricta* (Fig. 3A), supporting the use of pH 5.0 in subsequent acid treatment experiments. Given that the natural skin surface pH typically ranges from 4.5 to 5.5 (13), the undissociated form of AcOH may similarly enter fungal cells *in vivo*, potentially altering chromatin structure and gene expression in neighboring *Malassezia* populations.

Beyond regulating fungal responses, AcOH and other organic acids secreted by skin microbes can diffuse into host skin cells, potentially influencing histone modifications and gene expression. The skin, which serves as a barrier, is constantly exposed to microbial metabolites that may modulate the epigenetic landscape of host cells. AcOH, which induces acidic stress, has been shown to increase histone acetylation and alter chromatin structure in mammalian cells (29, 43). This modification may influence immune responses, as well as skin differentiation and barrier function, all of which are crucial for skin homeostasis and protection (29, 39, 40). However, the extent of these effects may vary depending on the local pH within different layers of the skin. While the outermost layers, such as the stratum corneum, stratum lucidum, and stratum granulosum, are acidic (known as the acid mantle), the deeper viable layers where living keratinocytes reside (e.g., stratum spinosum) maintain a near-neutral pH around 7.0 (44). Given that the membrane permeability of AcOH decreases at higher pH, the uptake of AcOH by keratinocytes *in vivo* may be limited. Therefore, the dramatic chromatin remodeling observed in *Malassezia* under acidic conditions may not occur to the same extent in host cells. Nevertheless, further exploratory studies into these interkingdom interactions, particularly in models that reflect the skin’s pH gradient, may provide new insights into therapeutic targets for skin disorders associated with microbial dysbiosis. Of note, the subtle yet distinct differences between the coculture and direct AcOH treatment conditions raise the possibility that factors other than AcOH may have contributed to the observed chromatin dynamics. They may include other bacterial metabolites, physical interactions between fungal and bacterial cells, or contact-dependent signaling pathways that are activated during interkingdom association. Further investigation is needed to clarify the contribution of these potential factors in shaping chromatin dynamics during microbial coexistence. While the current study was conducted in liquid media, the degree of host exposure to AcOH may differ *in vivo*, as the skin constitutes a solid and complex environment. Although agar-based culture systems could potentially better mimic the solid environment of the skin, technical limitations, such as cell detachment upon AcOH application or growth inhibition when AcOH was pre-mixed into the medium, ultimately prevented their use in this study. In this context, future *in vivo* models or organ-on-a-chip systems may help overcome these challenges and more accurately replicate skin-associated microbial interactions. Furthermore, the interaction between *S. epidermidis* and *M. restricta* may exert distinct and potentially synergistic effects on host cells, which could impact immune signaling, inflammation, or tissue repair in ways that extend beyond their individual activities. Therefore, investigating these interactions in skin models will be essential for understanding their clinical relevance.

Beyond regulating fungal responses, AcOH and other organic acids secreted by skin microbes can diffuse into host skin cells, potentially influencing histone modifications and gene expression. The skin, which serves as a barrier, is constantly exposed to microbial metabolites that may modulate the epigenetic landscape of host cells. AcOH, which induces acidic stress, has been shown to induce histone acetylation and chromatin decompaction in mammalian cells (ref2). This modification may influence immune responses, as well as skin differentiation and barrier function, all of which are crucial for skin homeostasis and protection (29, 40, 41). Such changes in chromatin structure may exacerbate inflammation and impair tissue repair, contributing to skin conditions, such as atopic dermatitis and acne. Further exploratory studies into these interactions may provide new insights into therapeutic targets for the treatment of skin disorders associated with microbial dysbiosis.

This study reveals a novel mechanism of interkingdom communication in the skin microbiome, wherein AcOH secreted by *S. epidermidis* acts as an epigenetic signaling molecule that reshapes the chromatin landscape and transcriptional output of *M. restricta*. These findings establish a previously unidentified connection between bacterial metabolism, fungal genome organization, and gene regulation. By mapping the first three-dimensional chromatin structure of *Malassezia* sp. and linking it to metabolite-driven histone modifications and transcriptional suppression, we provide a comprehensive view of how microbial cross-talk influences fungal adaptation. Understanding such microbial interactions at the chromatin level opens new avenues for exploring how environmental conditions and dysbiosis affect skin health*.* To further expand on these findings, future studies should address the dynamic nature and potential reversibility of AcOH-induced chromatin changes. For instance*,* time-course experiments following co-culture with *S. epidermidis* or direct AcOH treatment could elucidate the temporal progression of histone acetylation and chromatin remodeling. In addition, it will also be important to determine whether these changes are reversible upon removal of AcOH or restoration of neutral pH conditions. Such studies would clarify whether AcOH acts as a transient epigenetic signal or induces stable chromatin alterations contributing to long-term fungal adaptation. Furthermore, it remains to be explored whether shifts in *S. epidermidis* metabolic activity under pathological conditions contribute to skin diseases such as seborrheic dermatitis and atopic dermatitis by modulating the epigenetic landscape of *Malassezia* species. Ultimately, this study highlights the potential of bacteria-derived metabolites as modulators of fungal epigenetic states within the skin microbiome, underscoring the importance of interkingdom interactions in shaping chromatin architecture and transcriptional responses.

## Data Availability

The data have been deposited in the NCBI Gene Expression Omnibus (GEO) under SuperSeries accession GSE304083, which includes RNA-Seq, Hi-C, and ChIP-Seq datasets organized into SubSeries GSE304078, GSE304079, and GSE304082.

## SUPPLEMENTAL MATERIAL

Supplemental Material mbio.01592-25-s0001.docx Supplemental text and figures.

Supplemental Tables mbio.01592-25-s0002.xlsx Tables S1 to S5.

ASM does not own the copyrights to Supplemental Material that may be linked to, or accessed through, an article. The authors have granted ASM a non-exclusive, world-wide license to publish the Supplemental Material files. Please contact the corresponding author directly for reuse.
