# Supplementary material for: Acetic acid produced by Staphylococcus epidermidis remodels chromatin architecture and suppresses gene expression in Malassezia restricta
Source: mBio. 2025 Sep 12;16(10):e01592-25. doi: 10.1128/mbio.01592-25 (PMC12505993; doi:10.1128/mbio.01592-25)
Supplement: Supplemental Material — Supplemental text and figures. [file mbio.01592-25-s0001.docx]

**Supplemental Materials**

**Supplemental Methods**

**Supplemental Figures 1-6**

**Supplemental Methods**

**Hi-C data analysis**

In situ Hi-C data were processed as previously described using the rfy_hic2 package (<https://github.com/rafysta/rfy_hic2>) (34). To ensure accurate comparisons between samples, a total of 30,000,000 reads from the dataset was randomly subsampled using the “random_sampling_mapfile.sh” script in rfy_hic2.

Paired-end reads were aligned to the *M. restricta* genome (ASM329048v1) using an iterative alignment strategy in bowtie2 (version 2.4.4). Each read from the paired-end dataset was independently aligned rather than being aligned in the paired-end mode. The unmapped segments of partially mapped reads were trimmed and the remaining sequences were realigned. This iterative process was repeated until the read length was reduced to 25 bp or until the read could no longer be aligned. Filtering of valid Hi-C interaction reads and matrix generation were performed using the default parameters of rfy_hic2. Bins with zero read coverage in an entire row or column were imputed for missing values in the Hi-C contact map using the average of their adjacent bins. If adjacent bins were also missing, the mean of the four diagonally adjacent bins (lower-left, upper-right, lower-right, and upper-left) was used for imputation. If all surrounding bins were missing, imputation was not applied to prevent artificial biases. Border strength was calculated using ICE-normalized Hi-C matrices at a 5-Kb resolution as described by Kim et al. and Tanizawa et al. (18, 21).

To compare Hi-C matrices, the hicBuildMatrix function in HiCExplorer v3.7.5 was used with a bin size of 10 Kb (35). All subsequent tools used in the analysis are part of the HiCExplorer suite. The matrices were normalized to the sample with the lowest read count using the “--normalize smallest” option under the hicNormalize function. Following normalization, the matrices were refined using the “diagnostic_plot” and “correct” options under the hicCorrectMatrix function to remove unmapped bins or bins that exhibited abnormally high signals. After bin filtering, the final matrices from each sample were compared using the “--operation log2ratio” option under the hicCompareMatrices function. Finally, the matrices were visualized as plots using the “clearMaskedBins” option under the hicPlotMatrix function. Additionally, contact scores were measured as a function of genomic distance using the hicPlotDistVsCounts function with the “--maxDepth parameter” set to 20 million.

**Prediction of centromeric positions**

Inter-chromosomal interactions were extracted from the ICE-normalized Hi-C contact matrix of *M. restricta* at a 2-Kb resolution, and the total interactions for each 2-Kb bin were computed. To avoid strong telomere-telomere interactions, telomeres were defined as the terminal 40-Kb length of each chromosome and were excluded from the analysis. Of the remaining regions, bins exceeding the top 1% and 3% of total inter-chromosomal interactions were used as thresholds to define candidate centromeric regions. The 2-Kb bin with the highest interaction score was designated as the centromeric position (Fig. S1B).

**Measurement of contact score as a function of distance**

A distance curve was generated using intra-chromosomal interactions extracted from the ICE-normalized contact map of chromosome I at a resolution of 2 Kb. Genomic distances were transformed to a logarithmic scale by rounding log-transformed distances to two decimal places, and the mean interaction frequency was calculated for bins with the same transformed distance. For comparison of two samples, log2-transformed interaction scores were computed.

**Measurement of centromeric associations**

Hi-C matrices were loaded from .h5 files using the csr_matrix module in the SciPy package, and converted to the TSV format (36). The sum of contacts in each TSV file was computed and all datasets were normalized to the sample with the lowest total through the application of appropriate scaling factors. After normalization, for each centromeric bin, contact scores for bins located over 200 Kb away were summed based on the normalized TSV files. Then, these summed contact values per centromeric bin were compared across samples to evaluate differences in long-range centromeric interactions.

**Supplemental Figures**

**
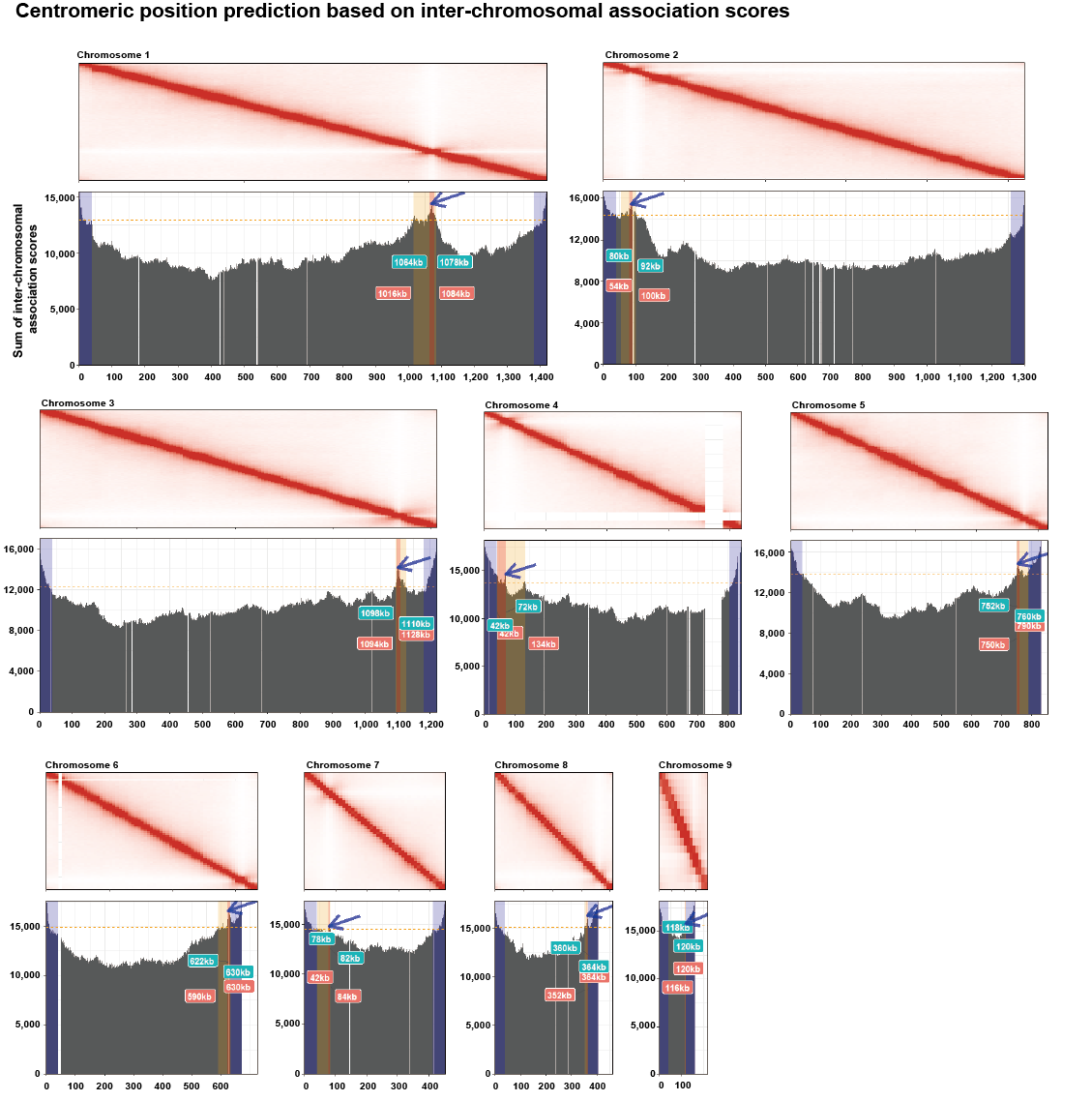
**

**Figure S1.** Estimation of centromeric positions in *M. restricta* based on inter-chromosomal association scores

Chromosome-wise Hi-C contact maps and inter-chromosomal association scores. Each panel displays a chromosomal Hi-C contact map (top) and the corresponding sum of inter-chromosomal association scores (bottom) for individual chromosomes (chr1-9). To identify candidate centromeric regions, the top 1% and 3% of inter-chromosomal association scores (excluding telomeric regions) were used as thresholds. Regions with scores exceeding the top 1% threshold are represented with red hatched boxes, while those with scores above the top 3% threshold are represented with yellow hatched boxes. Peaks in the inter-chromosomal association score are indicated with blue arrows. Genomic coordinates (red and blue labels) indicate the start and end positions of each predicted centromere based on the top 1% and 3% thresholds. Regions within 40 Kb of chromosomal ends, defined as telomeric regions, were excluded from the analysis and are represented with blue hatched boxes.


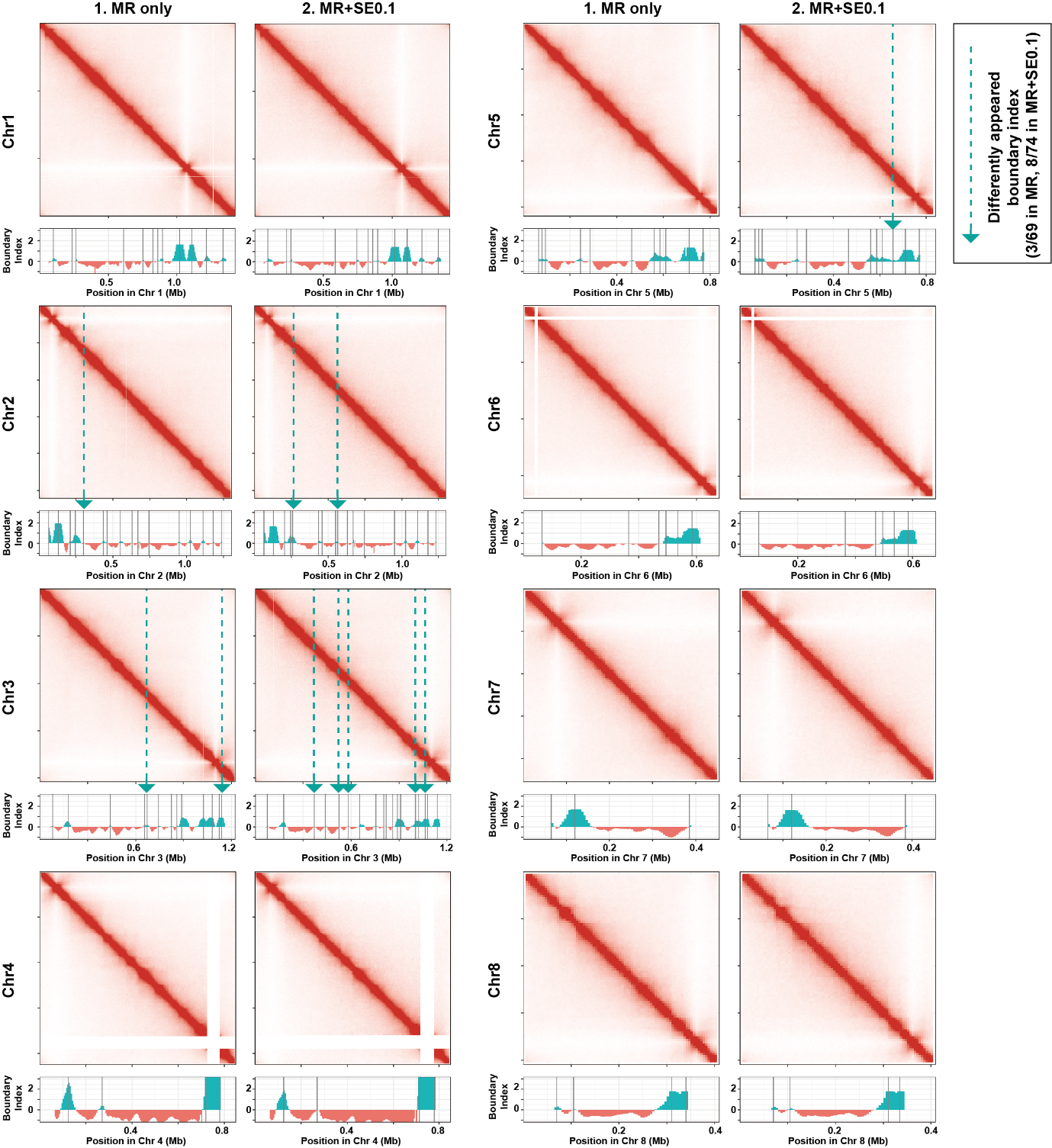


**Figure S2.** Genome-wide identification of TAD boundaries in *M. restricta* and their conservation under *S. epidermidis* co-culture

Hi-C contact matrices for each chromosome in *M. restricta* under axenic culture (MR only) and *S. epidermidis* co-culture (MR+SE0.1). Maps display intra-chromosomal interactions, with darker red shades indicating stronger interactions. The boundary index plots below each map highlight peaks (blue), which indicate TAD boundaries. In total, 69 TAD boundaries were detected under MR axenic culture, whereas 74 boundaries were identified under the MR+SE0.1 condition. Different TAD boundaries between the MR and MR+SE0.1 conditions are indicated using dashed arrows.


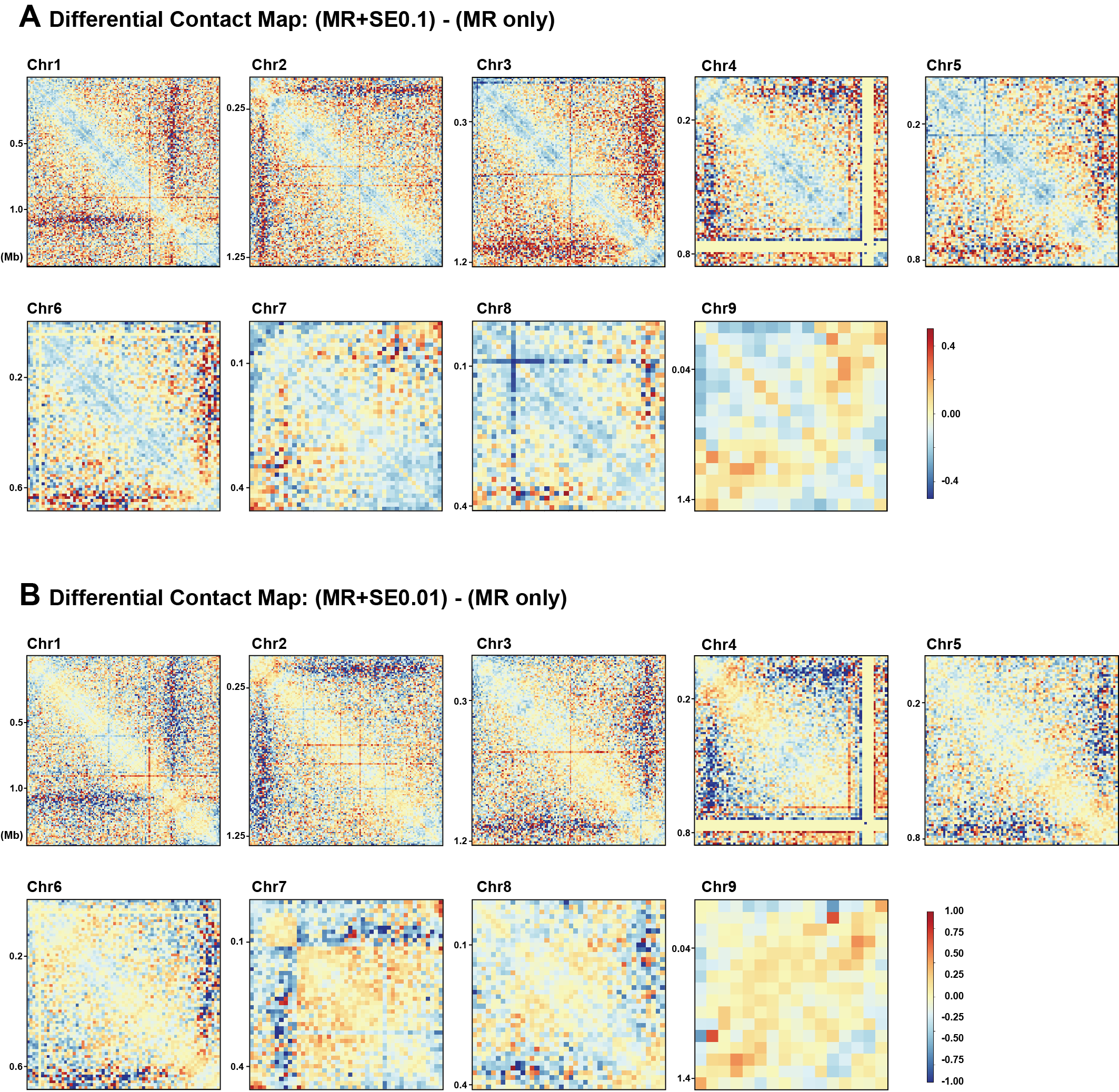


**Figure S3.** Chromosome-wide differential contact maps reveal *S. epidermidis*-induced chromatin remodeling in *M. restricta*

(**A**) Differential contact maps comparing the MR+SE0.1 condition to the “MR only” condition across all chromosomes. Blue regions indicate decreased chromatin interactions, while red regions indicate increased interactions. (**B**) Differential contact maps comparing the MR+SE0.01 condition to the “MR only” condition. Although the MR+SE0.01 condition also induced changes in chromatin interaction, its effect was less pronounced than that of the MR+SE0.1 condition, with fewer regions exhibiting a significant decrease in short-range interactions.


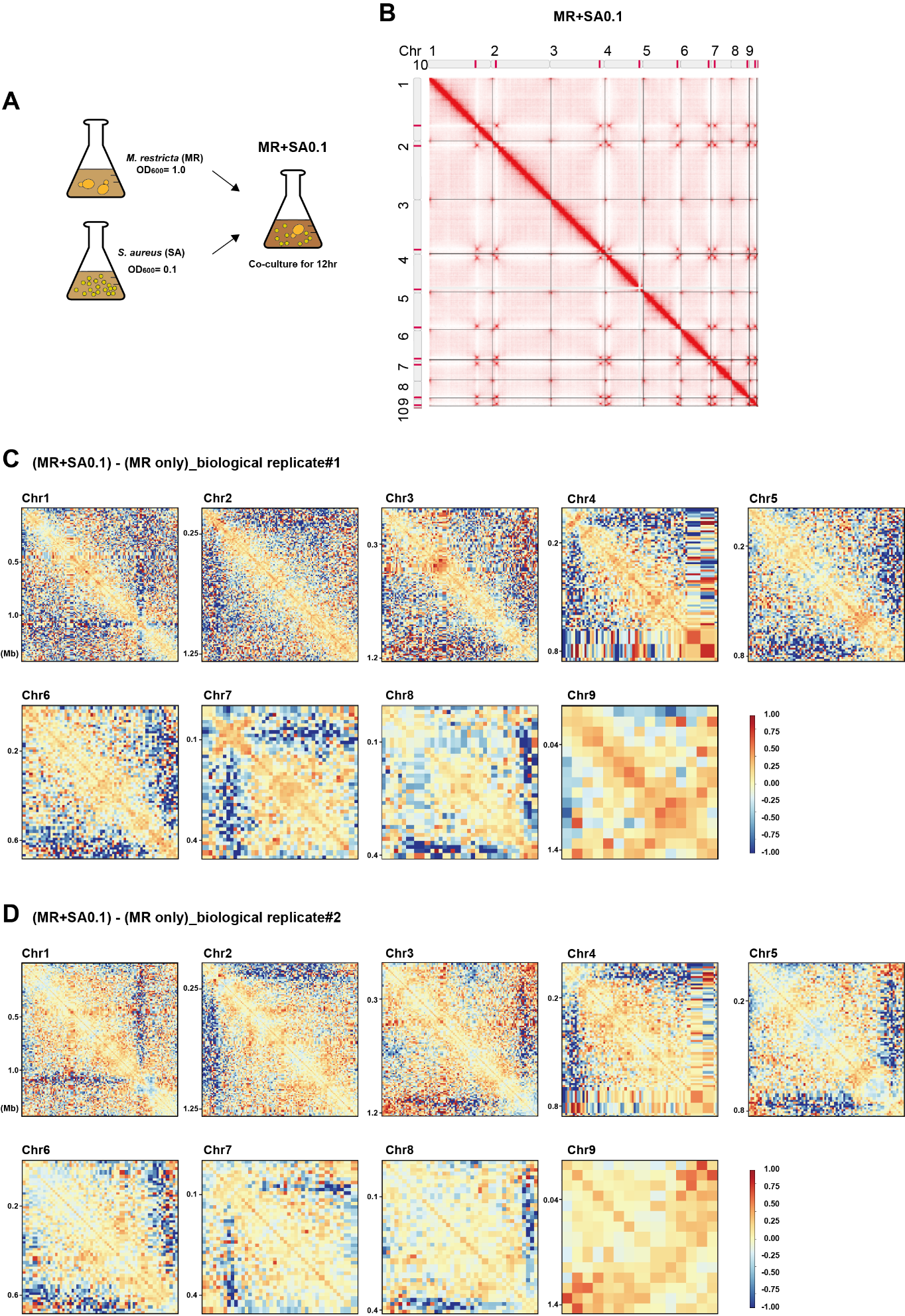


**Figure S4.** *M. restricta* maintains chromatin organization under *S. aureus* co-culture

(**A**) Experimental design for the in situ Hi-C analysis of *M. restricta* co-cultured with *S. aureus* (MR+SA0.1). (**B**) Genome-wide Hi-C contact map of *M. restricta* co-cultured with *S. aureus*. (**C**-**D**) Differential contact maps from two biological replicates comparing the MR+SA0.1 condition to the “MR only” condition.


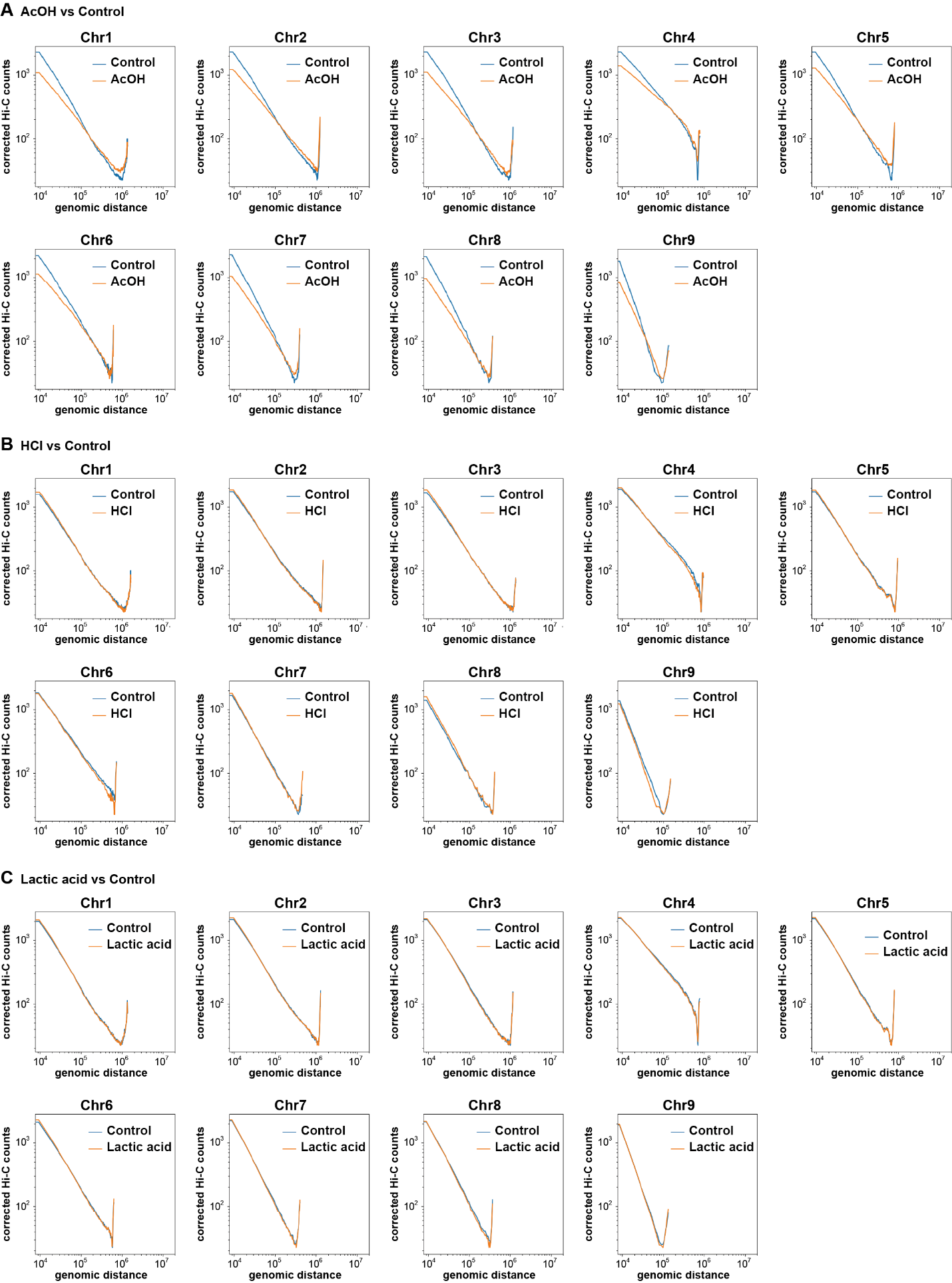


**Figure S5.** Chromatin contact probability curves for all chromosomes of *M. restricta* under acidic stress

Genome-wide Hi-C contact probabilities for all chromosomes (chr1-9) of *M. restricta* plotted against genomic distance under treatment with (**A**) AcOH, (**B**) HCl, and (**C**) lactic acid.

**
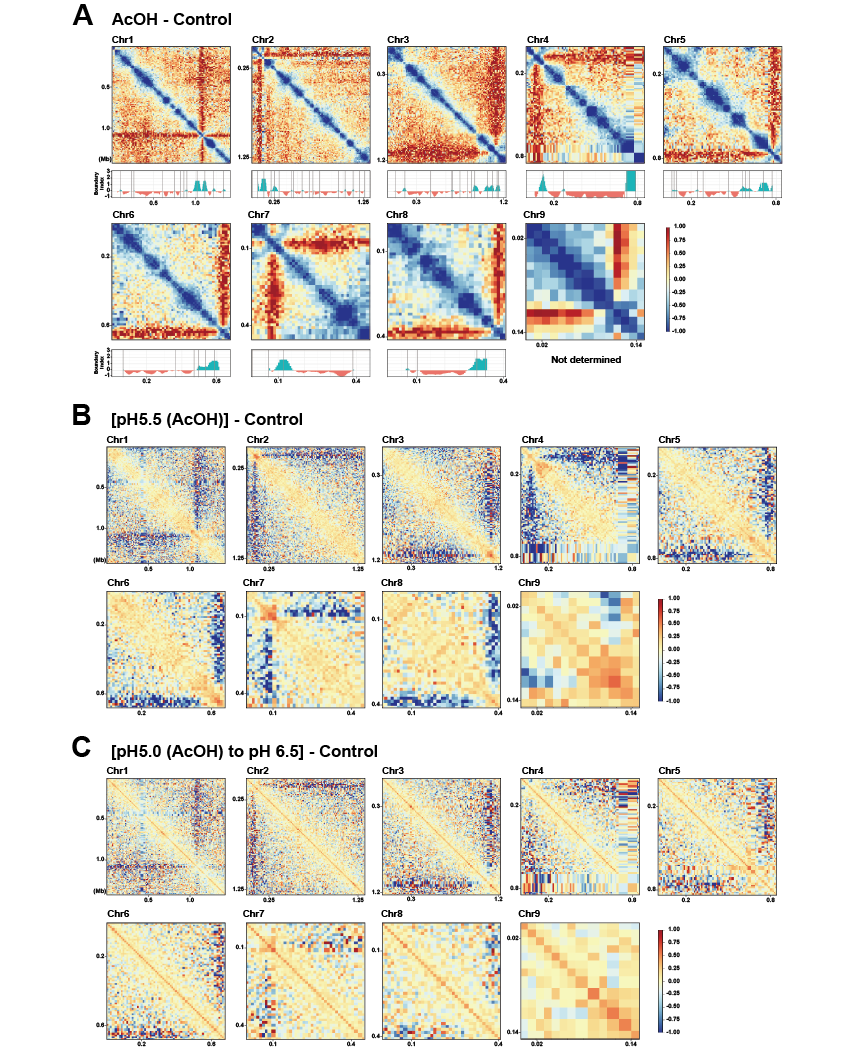
**

**Figure S6.** Genome-wide chromatin remodeling in *M. restricta* under acidic stress

(**A**) Differential Hi-C contact maps comparing acetic acid (AcOH)-treated to control conditions across chromosomes 1–9 in M. restricta. Regions with decreased interactions strongly correspond to topologically associating domains (TADs). (**B**, **C**) Differential contact maps comparing milder acidic conditions: pH 5.5 AcOH treatment (**B**) and AcOH treatment at pH 5.0 subsequently adjusted to pH 6.5 with NaOH (**C**) to control conditions across chromosomes 1–9 in *M. restricta*.
